# Emergence and evolution of social self-management of Parkinson’s disease: study protocol for a 3-year prospective cohort study

**DOI:** 10.1186/1471-2377-14-95

**Published:** 2014-05-02

**Authors:** Linda Tickle-Degnen, Marie Saint-Hilaire, Cathi A Thomas, Barbara Habermann, Linda S Sprague Martinez, Norma Terrin, Farzad Noubary, Elena N Naumova

**Affiliations:** 1 Department of Occupational Therapy, School of Arts & Sciences, Tufts University, Medford, MA, USA; 2 Department of Neurology, Parkinson’s Disease and Movement Disorders Center, Boston University Medical School, Boston Medical Center, Boston, MA, USA; 3 School of Nursing, College of Health Sciences, University of Delaware, Newark, DE, USA; 4 Department of Public Health & Community Medicine, School of Medicine, Tufts University, Boston, MA, USA; 5 Department of Medicine, School of Medicine, Tufts University, Boston, MA, USA; 6 Department of Civil and Environmental Engineering, School of Engineering, Tufts University, Medford, MA, USA

**Keywords:** Parkinson’s disease, Social participation, Daily life activities, Care giving, Facial expressiveness, Gender

## Abstract

**Background:**

Parkinson’s disease affects facial, vocal and trunk muscles. As symptoms progress, facial expression becomes masked, limiting the person’s ability to communicate emotions and intentions to others. As people with the disease live and reside in their homes longer, the burden of caregiving is unmitigated by social and emotional rewards provided by an expressive individual. Little is known about how adults living with Parkinson’s disease manage their social lives and how an inability to be emotionally expressive can affect social connections and health. Because social networks have been shown to be crucial to the overall well-being of people living with chronic diseases, research is needed on how expressive capacity affects life trajectories and health.

**Methods/Design:**

The overall objective is to understand the emergence and evolution of the trajectories of the self-management of the social lives of people living with Parkinson’s disease. The central hypothesis is that expressive capacity predicts systematic change in the pattern of social self-management and quality of life outcomes. The specific aims of this 3-year longitudinal study of 120 people with the disease and a maximum of 120 care partners are: 1) characterize social self-management trajectories over a 3-year period; 2) estimate the degree to which expressive nonverbal capacity predicts the trajectory; and 3) determine the moderating effect of gender on the association between expressive capacity and change in social self-management. Each participant will be assessed 14 times to detect rapid and non-linear changes in social participation and management of social activities; social network; and social comfort, general health and well-being.

**Discussion:**

This project will provide evidence to guide the development of interventions for supporting social integration of those living with Parkinson’s disease, thus leading to improved overall health. It focuses on the novel construct of social self-management and known factors—expressive capacity and gender—that contribute to stigmatization. The repeated measures design detects triggers of rapid changes in social and health outcomes.

## Background

Across the world, the increases in both life expectancy and independent living among older adults pose challenges for managing daily life and social participation [1]. People aging with disabling diseases such as Parkinson’s disease (PD) face these challenges to a greatly magnified degree. PD is one of the most common age-related neurodegenerative disorders. Its prevalence increases from approximately 554 per 100,000 in United States’ adults in their 60’s to 2,949 per 100,000 in those over 85 [2]. The disease is characterized by a progressive and variable rate of decline in speed, flexibility, fluidness and coordination of movement throughout the body. As the motor impairment of the disease affects facial, vocal, and trunk muscles, an *expressive mask* descends, significantly curtailing the person’s ability to express feelings, thoughts and intentions to others [3].

Muted expression in face, body or voice can negatively bias first impressions of a speaker’s character [3,4]. Both lay observers and health practitioners perceive highly masked women and men as having less desirable personalities, being less desirable social partners and friends, and showing less competence in performing emotional, social, and cognitive tasks of daily living than more expressive women and men with PD [5-8]. The stigmatization of masked expression is more severe for women than for men. Masking violates gender norms for women more than for men, because women are expected to be emotionally expressive and men to be stoic. People with PD can feel imprisoned in their unresponsive bodies and incapable of meeting minimal social norms of interpersonal behavior important for creating and sustaining relationships with family, friends, co-workers and others in their social environment [9].

### Parkinson’s disease and daily social life

Extensive research has shown that social engagement and supportive social networks protect the health and well-being of older adults [10-12]. Studies are small and rare that address how families manage their social lives and relationships while living and aging with PD and its progressive disability and complicated medical management [13-15]. Longitudinal studies of PD typically do not examine the dynamics and management of social life [16-19]. These studies do however provide initial evidence that people with PD become lonelier, more emotionally vulnerable and more socially isolated as the disease progresses, and show that changes in disability and socio-emotional well-being can be detected over a 3- to 4-year period. Family caregiving in PD can be emotionally and physically exhausting, yet some families report emotionally rich and rewarding lives [14,20-22]. Families living with a person who has greater capacity to express spontaneous warmth and gratitude may have a more enriched interpersonal and social life than families living with someone who is less capable of displaying positive feelings. No research, to the best of our knowledge, has addressed this possibility or the outcomes of expressive masking on physical and mental health in PD.

### A model for studying the social self-management of chronic disease

Most research on the management of daily living with PD has focused on self-management of physical symptoms by following practices such as doing exercises, taking medication, or modifying activities to conserve energy or prevent falling [23,24]. Yet our preliminary studies of daily living with PD have demonstrated that social concerns are a primary focus for individuals with PD and their care partners, that disease symptoms and social aspects of self-care are inter-related and mutually influential, and that social networks and environment are critical factors in navigating daily life with PD [25,26]. Expressive disability creates additional needs to manage stigma, public understanding and perceptions [27,28].

The current project proposes that *social self-management of chronic disease* is a valuable quality of life indicator. We define social self-management as the practices and experiences that ensure personal social comfort while supporting mental and physical well-being. Articulating this model will guide research to identify social factors that are deleterious to or protective of quality of life when living with chronic disease. PD offers a model for studying the effect of physical disease on the social self-management of daily life when physical symptoms affect fundamental social capacities. Table 1 shows our definition of social self-management and the contrasting and physically-focused definition of “looking after one’s health” in the World Health Organization’s International Classification of Functioning, Disability & Health (ICF) [29]. Our definition brings to the forefront the missing social link in the management of health.

**Table 1:** Definitions of social and physical self-management of health

**Type**	**Definition**
Social	Ensuring personal social comfort while supporting mental and physical well-being, such as
	– participating in valued social activities,
	– maintaining rewarding interpersonal relationships,
	– seeking help and support from capable people in informal and formal social networks.
Physical	Ensuring physical comfort, health and physical and mental well-being, such as
	– maintaining a balanced diet and an appropriate level of physical activity,
	– keeping warm or cool,
	– avoiding harms to health,
	– following safe sex practices, including using condoms, getting immunizations and regular physical examinations.

Note. The definition of social self-management is our own, while the definition of physical self-management is from ICF code d570, “Looking after one’s health”.

**Figure 1:**
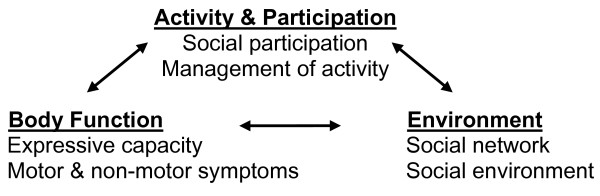
Social self-management unites multiple domains from the ICF as a conceptual unit.

To better understand the social lives of people with PD, it is necessary to translate motor impairment in the face, body and voice into social participation outcomes. A social ecological analysis of facial, bodily and vocal behavior produces this translation [30-32]. Healthy engagement in social life occurs in relation to bodily capacities and resources in the social environment of informal and formal social networks. Figure 1 shows that *social self-management* is a social ecological model that integrates each of three major ICF components of health together as a conceptual unit. Our longitudinal study of the daily lives of individuals with PD and their care partners is designed to validate the construct of social self-management as an evolving ecological system.

The objective of this project is to understand the social self-management systems and trajectories of people living with PD. The specific aims are as follows:

1. Characterize social self-management trajectories of individuals with PD over a 3-year period by assessing: a) social participation and management of social activities, b) informal and formal social networks, and c) social comfort and overall health and well-being. Hypothesis: Over three years there will be a general decline in the facilitative components of social living that comprise social self-management.

2. Estimate the degree to which expressive nonverbal capacity predicts the social self-management trajectory. Hypothesis: People with PD with higher expressive capacity at baseline will have more positive social trajectories over time than people with less expressive capacity.

3. Determine the moderating effect of gender on the association between expressive capacity in PD and change in social self-management. Hypothesis: Gender will moderate the association. The social trajectories of women are expected to be more vulnerable to the influence of expressive disability than the trajectories of men.

## Methods/Design

### Research design

This prospective cohort study design tracks general patterns of the sample as a whole as well as variation among individual trajectories in social self-management. The tracking creates evidence necessary to develop social life interventions that address the typical issues faced by people living with the disease as well as individual variations in needs. Over a 3-year period, the study follows 120 individuals with PD and their associated primary care partner. Individuals with PD are included whether or not they have an identified care partner.

**Figure 2:**
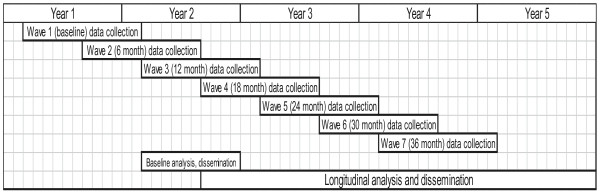
Timeline.

There are seven full assessments of approximately two hours duration and in-person, one at baseline and one every six months thereafter, with six in a clinical research lab and one in the home. Between the 6-month full assessments, there is an additional brief telephone call (15 to 30 minutes), totaling seven phone calls over three years. Our innovative design of 14 assessments over the three years aims at detecting social or health triggers that affect individual participant’s trajectories [33]. Frequent points of contact, while also building statistical power, increases the possibility of capturing remembered details of daily life that send health patterns into non-linear trajectories, such as anniversaries, retirement, bad colds or significant family events [34,35]. Figure 2 shows the study timeline.

### Ethics review

Recruitment, consent and data collection protocol have been approved by the Social, Behavioral & Educational Institutional Review Board of Tufts University Medford Campus (protocol # 1212038), and the Boston University Medical Campus (BUMC) Institutional Review Board (protocol # H-32114).

### Participants

#### *Recruitment*

PD is documented to be more prevalent in men than women: 1.55 men for every woman [2]. To maximize the power of our hypothesis testing of gender differences (Aim #3) the plan is to oversample women, by targeting a gender distribution of 50% women (n = 60) and 50% men (n = 60) with the disease and their care partners, a maximum combined total of 240 participants. We recruit participants through the BUMC Parkinson’s Disease Movement Disorders Clinic, postings on PD and aging research and advocacy websites, and PD support groups in the urban, suburban and rural regions within driving distance of the Boston metropolitan area.

Because people of color with PD often go undiagnosed in the United States and are less likely than their white counterparts to be engaged in disease-specific treatment groups and services [36,37], we have developed a plan to target enrollment among underrepresented groups. We use a grassroots recruitment strategy that employs a community-engaged approach to disseminate recruitment information and extend our referral network via community health centers, service organizations, housing services, religious centers/churches, grassroots leaders, and minority-serving local publications and media.

#### *Eligibility screening*

Individuals who respond to recruitment are screened in a 1-hour session by a movement disorders neurologist and movement disorders nurse specialist for eligibility and to collect demographic data and conduct a medical history protocol. Informed consent procedures are conducted prior to screening for eligibility, and consenting participants sign the BUMC consent form. Before their baseline assessment, participants sign their Tufts University consent form. Screening and assessments take place at the BUMC Parkinson’s Disease and Movement Disorders Center or at the Tufts University Health Quality of Life Lab in Medford.

Inclusion criteria for participants with PD ensure minimal capacity to participate in the study procedures:

1. Diagnosis of idiopathic PD utilizing the UK Parkinson’s Disease Society Brain Bank clinical diagnostic criteria, as evaluated by the neurological team,

2. Modified Hoehn and Yahr stage 1 through 4,

3. Score ≥ 26 on the Mini-Mental Status Exam,

4. Home setting within travel distance to study locations,

5. Able to communicate clearly and in English with research staff,

6. Interested in participating and willing and able to provide informed consent.

Inclusion criteria for care partners are very flexible since participation of a care partner, while desired, is not required:

1. Person with PD must consent for care partner to participate,

2. Score ≥ 26 on the Mini-Mental Status Exam,

3. Able to communicate clearly and in English with research staff,

4. Interested in participating and willing and able to provide informed consent.

### Baseline and 6-month follow-up in-person assessment procedures

Each of the seven in-person assessment sessions is designed to take a maximum of two hours to complete. At each session, initially, participants with PD and care partners are interviewed separately with parallel questionnaires to assess each of their daily life activities, health and quality of life. After separate interviews, the two are interviewed together about their combined social self-management. Participants with PD are asked to take their medication approximately 45 minutes before their assessment session in order to be “on”, that is, moving and functioning at maximal capacity during their session. Upon arrival they are asked about the timing and effectiveness of their medication on that day as well as the severity of their movement symptoms. Updates on medication changes and background information are collected such as, marital status, living arrangement, occupational status, and notable mental and physical health or life events in the recent past or since the previous visit.

### *Covariate measures*

The diminished expressive capacity that occurs in PD is a motor problem that can be confounded with the motor symptoms of depression, apathy or cognitive impairment [38]. At initial screening for this study, individuals with dementia are screened from participation (i.e., ≥ 26 on the MMSE) yet dementia may develop over the course of the study. We monitor dementia and basic cognitive functioning of both participants at every visit with the Montreal Cognitive Assessment [39]. The Geriatric Depression Scale [40] is administered to both participants at every visit to monitor depression throughout the study period. We do not exclude participants or data based on these measures. Rather we will use the measures for covariate analysis and to group individuals for blocked analyses.

### Primary interview measures

The order of assessments is designed to maximize rapport and coherence of the sequence of questions, while minimizing fatigue effects on performance assessments. Unless noted as otherwise, assessments are administered to both the participant with PD and the care partner. Variables and assessment procedures are described below in order of priority to our overall study objective and summarized in Table 2.

**Table 2:** Summary of assessments

**Assessment**	**ICF component**
**In-person full interview**	
Montreal Cognitive Assessment (MoCA)	B
Geriatric Depression Scale (GDS)	B
Activity Card Sort (ACS)	A
Qualitative self-management interview	A, B, E
Social network items	E
Chronic Illness Resource Survey (modified) (CIRS)	E
Home visit assessment^1^	E
Social Isolation domain, Nottingham Health Profile (NHP)	B, E
Positive Social Interaction items, Medical Outcome Study: Social Support Survey (MOS)	B, E
Stigma Scale for Chronic Illness (SSCI)	B, E
Version 2 of 12 item form of SF-36 (SF-12)	A, B
39 item Parkinson’s Disease Questionnaire^2^ (PDQ-39)	A, B, E
8 item form of PDQ-39^3^ (PDQ-8)	A, B, E
Movement Disorder Society Unified Parkinson’s Disease Rating Scales^4^ (MDS-UPDRS)	B
**Telephone interview**	
Short qualitative management interview	A, B, E
SF-12	A, B
PDQ-8^3^	A, B, E

Notes. All assessments are administered to both participants, except PDQ-39, PDQ-8 and MDS-UPDRS, which are administered only to the participant with PD. Unless otherwise indicated in footnotes, all in-person full interview assessments are administered at baseline and six months thereafter. All telephone assessments are administered at month 3 and every 6 months thereafter (between full interview assessments). Abbreviations: PD = Parkinson’s disease; ICF = International Classification of Functioning, Disability & Health, A = Activities & Participation, B = Body function, E = Environment; MOS = Medical Outcomes Study.

^1^The modified CIRS is the primary assessment for the home visit, along with an assessment of environmental barriers and facilitators of participation. Administered at month 18 only.

^2^Administered at baseline, at month 6 and every year thereafter.

^3^Administered when PDQ-39 not administered: at year 1 and every year thereafter.

^4^Administered at year 1 and every year thereafter.

### Social participation and management of social activities

Measures described here focus on assessing participants’ experience and participation in social activities and their self-management of social life – which fall under the Activity & Participation construct of the ICF -- and its relationship to their motor and non-motor symptoms— which fall under the Body Function construct of the ICF. These measures are administered at every visit.

#### Participation

The Activity Card Sort (ACS) [41] provides a measure of social participation and activity continuity from the past into the present. It contains photographs of individuals performing activities in four activity domains: 20 photographs of instrumental activities, 35 of low-physical demand leisure activities, 17 of high physical demand leisure activities, and 17 of social activities. The participants sort the cards into the categories of *never done, do less than six months ago, do same as six months ago, do more than six months ago,* and *given up the activity*. Scores are calculated for each domain and the total set of activities: 1) percentage of total retained activities, 2) percentage of retained activities that are performed less than six months ago, and 3) percentage of retained activities that are performed more than six months ago. The ACS demonstrates test-retest reliability in older adult community-living and clinical populations, is responsive to change in activities over a 6-month period [42] and correlates with mental and physical health in older adults [43]. It demonstrates convergent validity with the Adelaide Activities Profile [44], the PD-specific quality of life measure (Parkinson’s Disease Questionnaire-39) and the primary measure of PD symptoms (Movement Disorder Society Unified Parkinson’s Disease Rating Scales). It discriminates between activity patterns of men and women [45].

#### Management

An open-ended interview elicits participants’ reflections about self-identified frustrating and satisfying recent events in daily life and how they manage these and similar events [8,24,46]. Probes include 1) *What do you normally do to be able to participate in this or similar activities?* and 2) *What routines or strategies help you participate in this or similar activities?* Next, participants describe an activity outside of the home and how they get ready for it and manage PD symptoms to do it. Finally, participants are asked – *How would you rate your overall ability to manage participating in your daily life activities?* They provide a response on a scale of 1 (not at all effective) to 5 (highly effective). Participants with PD are videotaped and care partners are audiotaped.

In a second management discussion, we bring together the person with PD and the caregiver and ask them to think of an activity outside of the home that they recently did together. Probes include: 1) *How do the two of you get ready for an activity like this?* 2) *Do you think about timing of medication?* 3) *Are there symptoms you have to control when you go out?* and 4) *How do you manage doing both physical health activities, like taking medication, and engaging in social activities out of the home?* The discussion is videotaped with the camera focused only on the participant with PD.

### Social networks

Measures described here assess participants’ social environments, specifically informal and formal social network composition, contact frequency, social exchange of support, and the physical environments of social participation—which fall under the Environment construct of the ICF.

#### Social network composition, contact and exchange

At every visit, we use a set of items that discriminate cultural differences in social networks [47], including having a spouse or partner in the household, number and composition of individuals living in the household, total number of children and total number of grandchildren. Network contact is measured by frequency of contact with the most contacted child, with the most contacted friend, and with the care partner (if not the most frequently contacted child or friend). Social exchange is rated from 1 (very little) to 5 (a large amount) on 18 items. Nine of these items rate the amount of support received in three domains—help with daily activities, emotional support, and financial support—from each of three sources, if applicable: the other study participant (care partner or person with PD), other members of the household, and social network members outside of the household. The other nine items rate the amount of support given by the participant to the other care participant, other household members and others outside of the household, if applicable.

#### Social resources

At every visit, a modified version of the 22-item Chronic Illness Resource Survey (CIRS) assesses quality, composition and use of personal coping resources, informal social resources (e.g., family, friends, neighborhood, community) and formal social resources (health care team, work, organizations, and media/policy). There are nine sub-scores and a total score assessing resource support. We modified wording slightly to encompass resources commonly available to people with PD (e.g., Parkinson’s support groups). We created parallel forms for the participant with PD and the care partner and, to do so, changed the wording of “chronic illness” to “health management” to make the questionnaire applicable for both. The original CIRS measure was validated on two large samples of community-living adults with a variety of illnesses and met all standards for psychometric soundness [30,48]. It is sensitive to change over a 1-year period and has provided findings useful for developing interventions.

#### Home visit

One assessment, scheduled approximately 1.5 years into the study, is in the home of each participant with PD. We use the modified CIRS to facilitate an audiotaped conversation about potential facilitators and barriers in the home and neighborhood environment that may affect social self-management.

### Social comfort, health and well-being

These measures assess social self-management outcomes. The social comfort measures assess emotional well-being derived from one’s social networks and are associated with the Environment construct of the ICF. The health and well-being measures assess health quality of life, disease severity and impairments and are associated with the Body Function construct of the ICF.

#### Social comfort

At every visit, three measures assess social comfort: the Social Isolation Domain of the Nottingham Health Profile (NHP) [49], the Positive Social Interaction subscale items of the Medical Outcomes Study: Social Support Survey (MOS) [50], and the Stigma Scale for Chronic Illness (SSCI) [51].

• The Social Isolation Domain of the NHP is a 5-item measure of loneliness, difficulty with contacting people, difficulty getting along with others, and feeling like a burden [49]. We converted the original dichotomous yes/no scale to a more psychometrically sensitive and ecologically valid continuous measure. Participants rate their agreement with statements related to social isolation on a scale from 1 (extremely disagree) to 5 (highly agree). The domain score has been found to be responsive to change in PD over time [18]. It provides a measure of loneliness, which can predict motor decline and risk of death over one year in an older adult population including people with PD [52].

• We modified the wording of the three Positive Social Interaction items in the MOS Social Support Survey to identify positive interaction frequency with the care partner or the person with PD, rather than a non-specific “someone.” Our wording is: “How often is each of the following kinds of support available to you *from your partner* if you need it?” The original subscales were developed and validated on 2,987 patients and have high internal consistency and stability over time [50]. These items measure mutuality in the care relationship [53].

• The 24-item SSCI was developed based on focus groups with people experiencing chronic neurological disorders including PD and has two domains: felt stigma and enacted stigma [51]. Felt stigma items assess the emotional experience of stigmatization such as worry, embarrassment and self-blaming. Enacted stigma items assess the perception that people act differently toward the respondent: acting uncomfortable, being unkind, avoiding contact, and unfair treatment. The total score and subscores demonstrate psychometric soundness in cross-sectional validation studies. For our study, the original scale is used with the participant with PD. A modified form is used with care partners to identify how being with the person with PD affects their own felt and enacted stigma. For example, our wording (italicized) is “Because of *my partner’s* illness, I have felt left out of things”.

#### Health and well-being

The measures to assess health and well-being are the SF-12 (version 2) [54,55], the Parkinson’s Disease Questionnaire-39 (PDQ-39) [56] or the shorter form PDQ-8 [57], and the Movement Disorder Society Unified Parkinson’s Disease Rating Scales (MDS-UPDRS) [58]. The SF-12 (version 2) is the only one of these measures that is given to the care partner in addition to the person with PD.

• The 12-item SF-12 (version 2) is a highly used and cross-culturally validated survey of functional health and well-being that is a short form of the SF-36 [54,55]. It provides a norm-based score that can be used to compare the respondent against population level health. The SF-12 has been shown to be responsive to longitudinal changes in health. This measure is administered at every visit.

• The PDQ-39 assesses life concerns of individuals with PD [56]. It is composed of a summary index and eight domain scores—mobility, activities of daily living, emotional well-being, stigma, social support, cognitions, communication, and bodily discomfort. A higher score indicates a higher self-perceived frequency of quality of life and health problems in the past month that are due to the disease, with 0 indicatin*g never a problem* and 100 *always a problem*. The index and domain scores have adequate internal consistency, convergent validity with health status and quality of life measures, test-retest reliability, and responsiveness to intervention. The exception is the social support scale which has weak psychometric properties [59]. The PDQ-8 summary index is a short form of the PDQ-39 that is administered in place of the PDQ-39 for approximately half of the in-person assessments to allow for time to administer the MDS-UPDRS once per year. The PDQ-8 provides adequate psychometrics for detecting minimally important differences in change in health status of PD over a 1-year period [57].

• The MDS-UPDRS is a widely-used clinical assessment and research tool for assessing motor and non-motor symptom severity [58]. Parts I and II assess self-reported non-motor and motor aspects of daily living. Parts III and IV assess observed motor capacity and extent of abnormal movement and are the primary measures of motor symptoms for this project. This assessment is administered once per year.

### Brief telephone assessments

Calls are scheduled to interview the participant with PD and the care partner separately. The primary objective of these calls is to help inform findings relative to shifts in social trajectories. Participants have the opportunity to elect an alternate means of assessment, such as a mailed questionnaire, if a telephone assessment is perceived as burdensome. We and others [60] have found that telephone assessments that occur between scheduled in-clinic visits are welcomed by people with PD, who typically do not find them burdensome, and experience them as comforting, positive attention. The 15- to 30-min audiotaped protocol involves the following assessments:

1. Recent important life events, changes in physical and mental health, and medication changes since the previous assessment,

2. PDQ-8 (administered only to the participant with PD),

3. Social Isolation domain-NHP,

4. Three simple 5-point scale questions (1 = low, 5 = high), followed by open ended probes: 1) *How satisfying is your social life right now?* (probes: stressful or exciting changes in networks, finances, activities); 2) *How satisfied are you with managing the effects of PD on your life right now?* (probes: physical, social, emotional effects); and 3) *How satisfied are you with your health right now?* (probes: physical, social, emotional health), and

5. SF-12 (version 2).

### Measure of expressive nonverbal capacity

The Interpersonal Communication Rating Protocol: Individual Expressive Behavior (Parkinson’s Disease Version) (ICRP-IEB) [61] is used as the primary measure of the expressive capacity of participants with PD in videotaped discussions about management of social activities at baseline and 6-month follow-ups. This rating protocol employs a “thin slice” method, which links discrete behaviors (e.g., an upturned lip, a movement of the limb) into socially meaningful units (e.g., smiling, happiness, dominance) that are closely aligned with individuals’ social life outcomes (e.g. health or work success) [62-64]. The primary method is to extract short segments (thin slices) from a video or audiotaped social interaction, and have raters draw behavioral or social conclusions from the segments. For this project, 60-second clips are extracted from the videotapes at two standardized time points during the interview: first when the participant is asked to describe a frustrating activity, and again when asked to describe an enjoyable activity [46]. 60-second clips have been found to yield optimal accuracy-to-slice length ratio for making judgments of behavior [65]. Using the methods described in the ICRP-IEB manual, trained research assistants view the clips separately and rate the quality, intensity and frequency of expressive behavior on 20 discrete actions (e.g., smiling, gesturing, bodily movement, vocal tone) and PD symptoms (tremors and postural slouch) that observers use as cues to form judgments about a target individual’s emotions, thoughts, social motives and personality. Our previous studies provide evidence that expressive capacity in PD can be measured reliably and validly at this social level of analysis [46,66]. Expressive behavior composite scores are formed based on principal component analyses.

### Power calculation

The data analysis assumes 120 participants with PD and seven full assessment time points over three years; dropout per year at 15%; intra-class correlation (ICC) at 0.82, based on prior data [24]; and type I error at alpha = 0.05. We have 80% power to detect a mean change over time of 0.40 of a standard deviation in the PDQ-39 outcome per year; 80% power to detect a correlation between continuous variables of 0.23; and 80% power to detect a difference of 0.50 standard deviations in the outcome scale between genders (Aim 3). Previous studies of similar outcomes have found a sample size of 120 to be adequate for demonstrating statistically significant effects [19,24].

### Data analysis

#### Aim 1: Social self-management trajectories

##### Descriptive analyses

Descriptive statistical analyses will be performed on the total sample and on demographic subgroups that are relevant to the progression of Parkinson’s disease (e.g., current age, age of onset, disease severity at baseline, gender). We will plot mean trajectories over time for all repeated measures by the same demographic subgroups. Scales will be checked for floor and ceiling effects. Associations between the scales, which represent the different components of social self-management, will be examined through scatter plots and relational statistics.

Audiotaped open-ended discussions during in-person and telephone assessment sessions will be transcribed. Quantitative content analysis will be performed on the transcriptions using Linguistic Inquiry Word Count (LIWC) to describe proportions of verbal content meaningfully related to the three ICF social self-management categories in our model (Figure 1) [67]. LIWC contains word dictionaries that measure 80 language dimensions including psychological constructs (e.g. affect, cognition), biological processes (e.g. body, health), personal concerns (e.g., social, home, work, leisure) and linguistic indicators of socio-emotional experience and interpersonal interaction. The dimensions have been validated in over 120 published studies as indicative of a variety of life preferences and health outcomes [68] and in our previous work on motivation indicators (e.g., helplessness, hopefulness, apathy) in PD [46]. Verbal content proportions from the LIWC analysis will be included with quantitative questionnaire results that are entered into quantitative descriptive and longitudinal analyses.

In addition to quantitative content analysis, qualitative content analysis will be performed on the transcribed open-ended narratives using standard methods [69]. Each transcribed interview will be searched for phrases meaningfully related to the three ICF social self-management categories in our model. We will code phrases and identify themes across participants that can be abstracted to these categories and the relationships between the categories (e.g. phrases that link a social activity to motor symptoms). If the data reveal new dimensions to social self-management, we will develop new codes, themes or categories to accommodate these data. To assess and assure coding reliability and dependability, two independent coders will assess a sub-set of data (approximately 40 transcripts of the same type) in an iterative process until coding agreement is achieved. Data will be summarized with coding categories and illustrated with participant quotations. We will compare and contrast qualitative and quantitative data to elaborate the construct of social self-management.

### Models for longitudinal trajectories

We will perform longitudinal data analysis using the multilevel model for change (also called random coefficient, mixed, or hierarchical model) [70]. Primary outcomes measured at baseline and the semi-annual fixed time points are: 1) retained activities (ACS), 2) proportions of quantitatively content coded words during open-ended responses of social self-management discussions (LIWC), 3) network composition, structure and exchange scores, 4) social network resource utilization scores (modified CIRS), 5) social isolation score, 6) positive interaction with partner score, 7) felt stigma and enacted stigma scores (SSCI), 8) physical and mental health scores (SF-12), 9) health quality of life with PD scores (PDQ-39 or PDQ-8), and 10) motor capacity scores (MDS-UPDRS, Parts III and IV). Outcomes measured at all 14 time points are: 1) physical and mental health scores (SF-12), 2) health quality of life with PD score (PDQ-8), and 3) loneliness score (Social Isolation, NHP). We will explore the temporal dynamics of these outcomes and will attempt to better understand the similarities, differences and factors governing such dynamics. By including random effects, we will explore the population trajectory and the degree of variations of individual trajectories. For outcomes reported by both the person with PD and the care partner, we will be depicting both perspectives, which enable us to assess the synchronicity between the two sources. We will formally evaluate, through statistical tests of interaction terms, whether particular factors affect the ratings of one source more than another.

The multilevel models can accommodate subjects with and without care partners, as well as incomplete data collection, as long as data are missing at random [71]. For sensitivity analyses we will use pattern mixture models to account for the possibility of informative missingness. The models will stratify by pattern of and reason for missing data [72].

### Effects of triggers on trajectories: Exploratory analysis

For exploratory purposes, we will expand the general multilevel modeling approach to consider potential effects of unanticipated triggers assessed at the individually identified time points [33]. We will re-calibrate the timing of measurements as “time elapsed since an event” or “time preceding an event”. Recalibration will allow us to synchronize triggering events across the cohort, allowing us to determine if there are synchronized changes in one of our outcomes of interest after occurrence of an unanticipated event. For example, there may be an increase in loneliness following hospitalization of a spouse or a decline in social activity following an episode of physical illness.

If a sufficient number of subjects have experienced a similar event, we will explore non-linearities with non-parametric fitting. For example, some social triggers may have a temporary U-shaped trajectory. This description of potential rapid or unexpected changes in the trajectories is highly innovative for research in the social aspects of PD and other chronic degenerative conditions. Adding trigger event monitoring to our research design builds power for repeated measures, and it may illuminate our understanding of disease progression as a social ecological phenomenon.

#### Aim 2: Expressive nonverbal capacity as predictor

We will start with a simple linear model, considering a random intercept and slope for each individual. We assume that, the variation among individuals in the intercept and slope of their trajectories can be explained by subject characteristics, including expressive capacity at baseline. For example, we may find that the rate of change in the study outcomes may vary and depends on expressive capacity at baseline. This preliminary step will guide the further model building.

We will build multivariate models by first adding demographic factors, then clinical factors, and then other variables of interest including masking, life stage, and access to support. We will reduce the number of variables in each group before adding the group to the model, and will reduce the number of variables further as groups are added. Since expressive masking will be measured at follow-up times as well, we will also perform repeated measures analyses to estimate the relation between change in expressive masking and change in social self-management.

#### Aim 3: Moderating effect of gender

We will apply models developed for Aim 1 and 2, and explore in details the effect of gender on the modeling results. For example, we may find that rates of change in the study outcomes depend on both, expressive capacity at baseline and gender. We will test the moderating effect of gender on the relationship between expressive masking and social self-management in PD by stratifying the analysis and by including an interaction term between gender and potential predictors. For all models, we will test for non-linearities in predictors and will transform variables when necessary to obtain good model fit. Models will be examined with respects to over-fitting and fine-tuned to improve their performance.

## Discussion

Our work has the potential to significantly advance PD research and evidence-based neurological nursing and rehabilitation by establishing the natural evolution of the social lives of people with PD and their families and its relationship to health outcomes. Uniquely, the study is designed to identify non-linearity in life trajectories that are triggered by social and health events. The proposed work develops the new construct of social self-management, and does so in a manner that reflects the daily lived experience of PD and fundamental factors, such as expressive capacity and gender, that have a demonstrated impact on interpersonal interaction. Our approach departs from the status quo in PD research in particular and chronic disease research in general by contributing an ecologically valid construct and an intensive repeated measures design for studying changes in chronic disease management and life outcomes.

We expect to find that people living with PD attempt to continue their valued activities while experiencing a decline in social participation as day-to-day management of the disease becomes more effortful and eventually less effective. However, very little is known about social self-management or its effectiveness in moderating decline.

Although social networks may suffer in general, relationships may be preserved and flourish in some individuals. Concomitant with the overall prediction, we expect to see a general decline in health and well-being. We expect that baseline expressive capacity will predict social trajectories of people living with PD. Those individuals with higher expressive capacity are expected to make their feelings and motives understood better and more rapidly than those with less capacity, allowing for less stigmatizing interaction and preserving their socially comforting lives. This effect is expected to be strongest for women due to gender norms related to expressiveness.

### Problems and alternative strategies

Attrition can be a problem in longitudinal studies of a disease like PD, due to morbidity, mortality or other uncontrollable factors. Our previous research, which enrolled similar numbers of people with PD, had a relatively low attrition rate despite significant time burden [24], and we expect a similarly low rate in the proposed study. We will collect data on reasons for any attrition, implement rigorous strategies to counteract controllable sources of attrition and test whether our strategies are effective. Retention strategies, such as activities that develop strong rapport with participants, may create unintentional “social intervention” effects on study outcomes [73]. We will apply our strategies with the intent of maximizing retention while minimizing and monitoring unintentional effects on everyday social life.

Other symptoms of PD may be more predictive of social outcomes than expressive masking. We will monitor other potentially stigmatizing symptoms, including tremor, drooling and stooped posture. In addition, we will augment and triangulate our ICRP-IEB measurement of spontaneous expression with a recently developed measure that specifically identifies discrete facial actions of participants with PD during a set of brief standardized emotion role-playing and imitation scenarios [74].

Although existing research is supportive of our hypothesis that gender moderates the effects of expressive capacity on life trajectories, there are many parameters of living with PD that cannot be controlled. Expressive masking may create vulnerability in women, but in general aging women are more likely than aging men to have strong social support networks, which is a possible confound for our study [75]. Recent literature suggests that PD presents and progresses differently in men and women, for example, that women may have a more benign disease than men [76]. Gender research into quality of life domains is in its infancy, thus our study is unusual and does not have precedents to follow. We will be careful to evaluate gender at all steps of the project.

### Long-term goal

Our long term goal is to guide the development of family-centered and evidence-based interventions aimed at supporting social integration and preventing isolation and loneliness in women and men living with PD. Interventions that support the social lives of people living with PD are likely to have a significant positive effect on both their and their family members’ physical health and socio-emotional well-being. To the best of our knowledge, this project is the first to address whether an individual’s capacity for nonverbal expression has a crucial predictive role in the social self-management of PD.Our intensive repeated measures design provides a model for detecting triggers that rapidly change life trajectories. We expect that the results will contribute solid evidence to guide the development of interventions that directly target protective and risk factors for the social self-management of PD. In addition, this project will provide a model for rigorous examination of social self-management in other chronic health conditions that affect middle-aged and older adults’ social capacity, such as other neurodegenerative disorders, stroke, facial paralysis, and hearing or visual loss.

## Competing interests

The authors declare that they have no competing interests.

## Authors’ contributions

LT conceived of the study and the overall study design, and was responsible for reporting and leading preliminary projects and drafting the manuscript. MS and CAT participated in conceiving and designing the study and took lead roles in developing details of clinical recruitment, screening procedures and sample size. BH consulted on concepts, literature and design aspects of the study related to family caregiving and qualitative methods. LSSM took the lead role in designing the grassroots community procedures for recruiting underrepresented populations. NT consulted on the aims of the study and contributed to the sample size determination and the development of the statistical analysis procedures. FN participated in developing the statistical analysis procedures. ENN contributed to the conception of the study and the sample size determination, and took a lead role in determining the number of assessment sessions for detecting non-linearity in life trajectories, and creating the plan for statistically modeling these trajectories. All authors read and provided feedback for the manuscript draft and approved the final manuscript.

## Authors’ information

LTD, PhD, OTR/L, FAOTA: Professor of Occupational Therapy, Director of Tufts Health Quality of Life Lab. MSH, MD, FRCPC: Associate Professor of Neurology, Medical Director of Boston University Medical Center Parkinson’s Disease and Movement Disorders Center. CAT, RN, MS, CNRN: Program Director of Boston University Medical Center Parkinson’s Disease and Movement Disorders Center. BH, PhD, RN, FAAN: Nannie Longfellow Professor of Nursing, Assistant Director of Research. LSSM, PhD: Assistant Professor of Public Health & Community Medicine. NT, PhD: Associate Professor of Medicine. FN, PhD: Assistant Professor of Medicine. ENN, PhD: Professor of Civil and Environmental Engineering, Associate Dean for Research.

## Pre-publication history

The pre-publication history for this paper can be accessed here:

http://www.biomedcentral.com/1471-2377/14/95/prepub
